# Supplementary material for: The interactome of intact mitochondria by cross-linking mass spectrometry provides evidence for coexisting respiratory supercomplexes
Source: Mol Cell Proteomics. 2017 Dec 8;17(2):216–32. doi: 10.1074/mcp.RA117.000470 (PMC5795388; doi:10.1074/mcp.RA117.000470)
Supplement: Supplemental Data [file supp_RA117.000470_133922_0_supp_23978_fzffwf.zip › spectra_annotation/mito_DR_spectra_annotation/256-1-5-1-1-1.pdf]

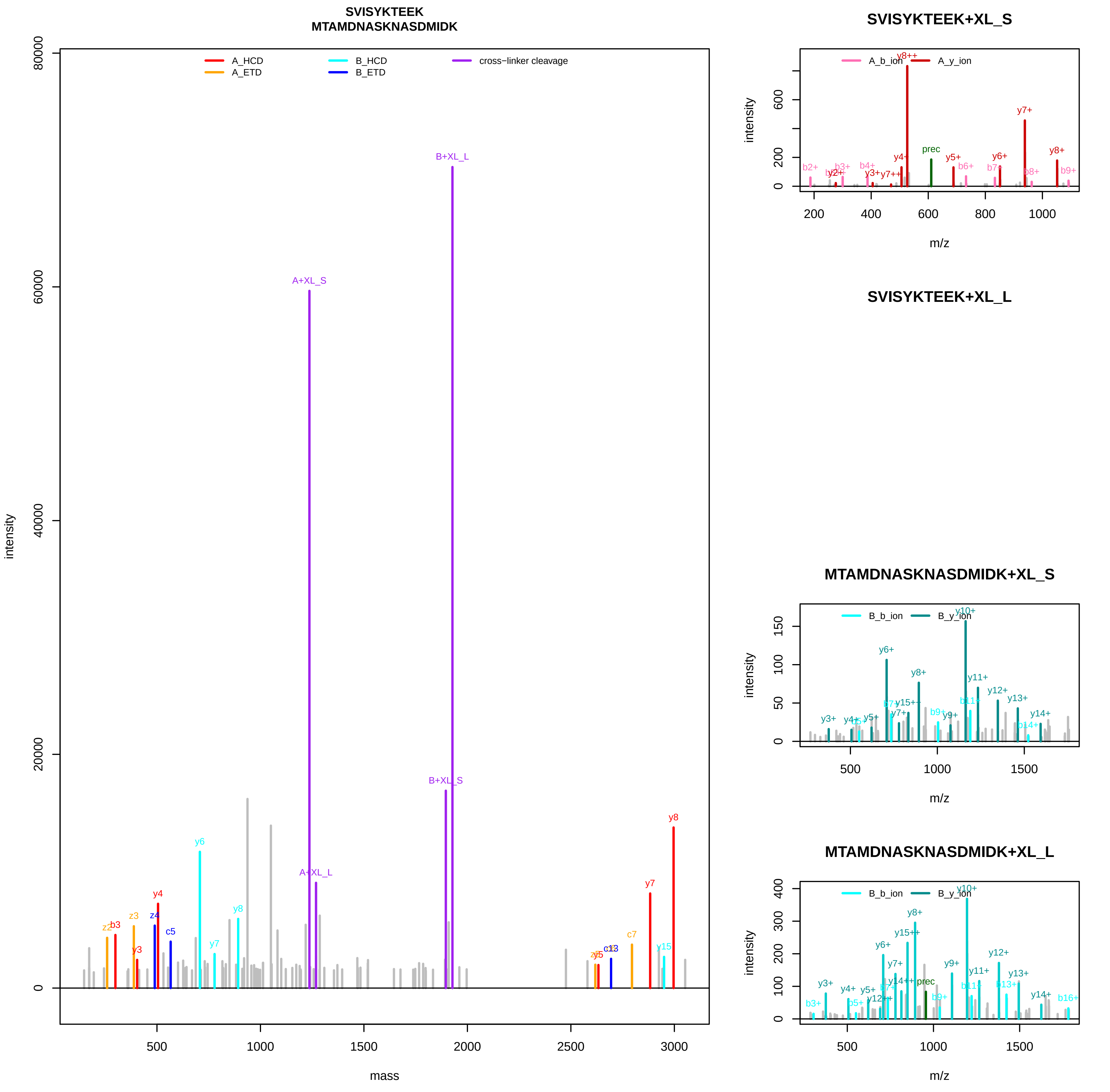
SVISYKTEEK
MTAMDNASKNASDMIDK
A_HCD
A_ETD
B_HCD
B_ETD
cross−linker cleavage
B+XL_L
A+XL_S
B+XL_S
A+XL_L
y8
y7
y6
y8
y7
y4
b3
z2
z3
y3
z4
c5
c7
c13
y15
y5
intensity
mass
SVISYKTEEK+XL_S
A_b_ion
A_y_ion
y8++
y7+
prec
y8+
y4+
y5+
y6+
b2+
b3+
b4+
b6+
b7+
b8+
b9+
y2+
y3+
y7++
m/z
SVISYKTEEK+XL_L
MTAMDNASKNASDMIDK+XL_S
B_b_ion
B_y_ion
y10+
y6+
y8+
y11+
y12+
y13+
y14+
y3+
y4+
y5+
y7+
y9+
y15++
b9+
b11+
b14+
MTAMDNASKNASDMIDK+XL_L
y10+
y8+
y15++
y6+
y7+
y12+
y11+
y9+
y13+
y14+
b16+
y3+
y4+
y5+
y12++
y14++
prec
b13+
b9+
b3+
b5+
